# Identification of an arabinopyranosyltransferase from *Physcomitrella patens* involved in the synthesis of the hemicellulose xyloglucan

**DOI:** 10.1002/pld3.46

**Published:** 2018-03-13

**Authors:** Lei Zhu, Murali Dama, Markus Pauly

**Affiliations:** ^1^ Department of Plant and Microbial Biology University of California Berkeley CA USA; ^2^ Institute of Plant Cell and Biotechnology University of Dusseldorf Dusseldorf Germany

**Keywords:** arabinopyranosyltransferase, glycosyltransferase, hemicellulose, *Physcomitrella patens*, xyloglucan

## Abstract

The hemicellulose xyloglucan consists of a backbone of a β‐1,4 glucan substituted with xylosyl moieties and many other, diverse side chains that are important for its proper function. Many, but not all glycosyltransferases involved in the biosynthesis of xyloglucan have been identified. Here, we report the identification of an hitherto elusive xyloglucan:arabinopyranosyltransferase. This glycosyltransferase was isolated from the moss *Physcomitrella patens*, where it acts as a xyloglucan “D”‐side chain transferase (XDT). Heterologous expression of *PpXDT* in the *Arabidopsis thaliana* double mutant *mur3.1 xlt2*, where xyloglucan consists of a xylosylated glucan without further glycosyl substituents, results in the production of the arabinopyranose‐containing “D” side chain as characterized by oligosaccharide mass profiling, glycosidic linkage analysis, and NMR analysis. In addition, expression of a related Physcomitrella glycosyltransferase ortholog of *PpXLT2* leads to the production of the galactose‐containing “L” side chain. The presence of the “D” and “L” xyloglucan side chains in the Arabidopsis double mutant *Atmur3.1 xlt2* expressing *PpXDT* and *PpXLT2*, respectively, rescues the dwarfed phenotype of untransformed *Atmur3.1 xlt2* mutants to nearly wild‐type height. Expression of *PpXDT* and *PpXLT2* in the *Atmur3.1 xlt2* mutant also enhanced root growth.

**Abbreviations**
2‐Xyl*p*: 2‐xylopyranose
AIR: Alcohol insoluble residue
CaZy: Carbohydrate‐active enzyme
GC‐MS: Gas chromatography mass spectrometer
GT: Glycosyltransferase
HPAEC‐PAD: high‐performance anion‐exchange chromatography with pulsed amperometric detection
MALDI‐TOF: Matrix‐Assisted Laser Desorption/Ionization Time of Flight Mass Spectrometry
OLIMP: Oligosaccharide mass profiling
t‐Ara*f*: terminal arabinofuranose
t‐Ara*p*: terminal arabinopyranose
XDT: Xyloglucan D‐side‐chain Transferase
XEG: Xyloglucan‐specific endoglucanase
XyG: Xyloglucan

## 1 INTRODUCTION

The plant cell wall is a complex extracellular matrix composed of polysaccharides such as cellulose, hemicellulose, various pectic polysaccharides, glycoproteins, and the polyphenol lignin. The major hemicellulose xyloglucan (XyG) is found in all land plants and is especially abundant in the primary cell wall of dicots (Pauly & Keegstra, 2016). XyG in the primary cell wall attaches to cellulose microfibrils noncovalently via H‐bonds, and its metabolism in the wall is thought to play a role in cell elongation (Keegstra, Talmadge, Bauer, & Albersheim, 1973; Somerville et al., 2004; Valent & Albersheim, 1974). However, the precise molecular role of XyG in plant growth and development is not clear (Park & Cosgrove, 2015; Talbott & Ray, 1992; Thompson, 2005) as mutant plants lacking XyG do not exhibit an obvious growth phenotype (Cavalier et al., 2008). Initially, it was thought that a particular XyG structure is plant‐specific, but recently tissue‐specific structures within a species have emerged (Dardelle et al., 2015; Lampugnani et al., 2013; Liu, Paulitz, & Pauly, 2015; Schultink, Liu, Zhu, & Pauly, 2014). XyG has not only been found in higher plants, but also in nonvascular plants such as liverworts and mosses (Pena, Darvill, Eberhard, York, & O'Neill, 2008).

XyG consists of a backbone of β‐1,4 glucan substituted with xylosyl residues that are often further decorated with other sugar residues and/or acetyl‐residues. More than 20 structurally different XyG side chains have been discovered to date (Pauly et al., 2013; Scheller & Ulvskov, 2010; Schultink et al., 2014), and their structural diversity can be described using a one‐letter code (Fry, Aldington, Hetherington, & Aitken, 1993). G refers to an unsubstituted glucosyl backbone residue, while X depicts a xylosylated glucosyl residue as in α‐D‐xylose‐6‐β‐D‐glucose. X can be further extended on the xylosyl unit at *O‐2* with galactosyl‐, arabinopyranosyl‐, galacturonsyl‐, xylosyl‐, or arabinofuranosyl residues resulting in L, D, Y, U, and S side chains, respectively (Hilz et al., 2007; Hsieh & Harris, 2012; Jia, Cash, Darvill, & York, 2005; Pena, Kong, York, & O'Neill, 2012; Pena et al., 2008).

**Figure 1:**
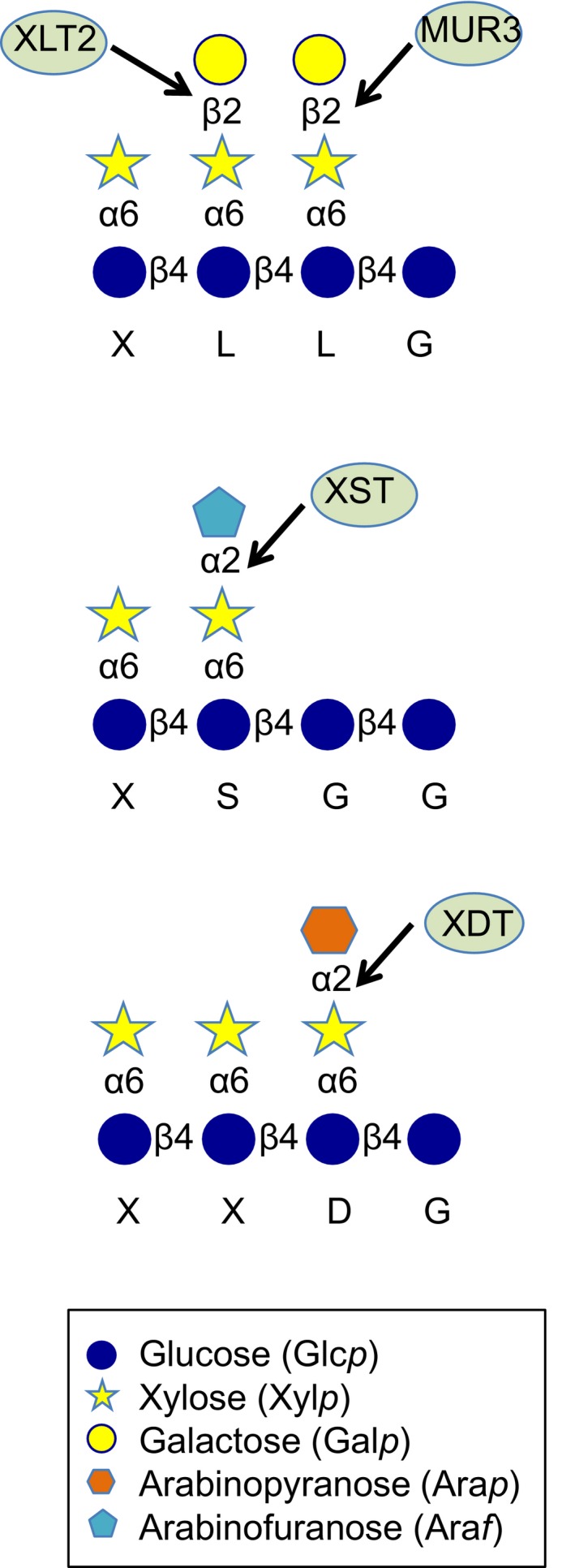
Xyloglucan oligosaccharide structures and GT47 glycosyltransferases involved in its synthesis. The xyloglucan one‐letter code nomenclature is indicated below the structure. MUR3 and XLT2 ‐ XyG:galactosyltransferases; XST ‐ XyG:arabinofuranosyltransferase; XDT ‐ XyG:arabinopyranosyltransferase

XyG assembly requires various glycosyltransferases (GTs) that add specific sugars to the extending polymer. Many GTs involved in XyG synthesis have been identified that belong to various carbohydrate‐active enzymes (CaZy) families based on gene‐sequence homology (Pauly & Keegstra, 2016; Coutinho et al., 2003). The GT47 family is involved in XyG side chain biosynthesis and includes MUR3, XLT2, and XST (Figure 1). MUR3 represents a XyG:galactosyltransferase, which adds a β‐galactosyl‐residue at the *O*‐2 position to a xylosyl residue resulting in the side chain L (Madson et al., 2003). MUR3 transfers the galactosyl moiety to a specific xylosyl residue on the XyG chain leading to the occurrence of an XXLG oligosaccharide motive in XyG. In contrast, a related GT47 protein, XLT2, adds the galactosyl‐residue to another xylosyl residue leading to a XLXG motive, indicating that these GTs exhibit regioselectivity (Jensen, Schultink, Keegstra, Wilkerson, & Pauly, 2012). GT47 family members can also transfer galacturonic acid (XUT1) or arabinofuranosyl moieties (XST) (Pena et al., 2012; Schultink, Cheng, Park, Cosgrove, & Pauly, 2013) to the xylosyl residue.

The moss *Physcomitrella patens* was found to contain XyG (Pena et al., 2008), with galacturonosyl and arabinopyranosyl residues at the *O*‐2 position of their xylosyl side chains (Pena et al., 2008). The arabinopyranosyl residue is unique as it has also been found in the XyG of lower plants such as the lycophytes including Selaginella, Equisetales, Polypodiales and Cycadales (Hsieh & Harris, 2012; Pena et al., 2008), but not in any gymnosperm or angiosperm plant to date.

To gain insights into the function of the arabinopyranosyl residue on XyG side chains, we describe the identification of the responsible arabinopyranosyltransferase in the Physcomitrella GT47 family. Because the simplest XyG side chain containing an arabinopyranosyl residue has been abbreviated with the one‐letter code D (Fry et al., 1993; Pauly & Keegstra, 2016), we named the responsible protein PpXDT (XyG D side chain Transferase).

## 2 METHODS

### 2.1 Plant growth

Seeds of Arabidopsis wild‐type Col‐0, *mur3.1 xlt2* (Schultink et al., 2013) and the transgenic plants generated here were germinated either in soil pots, or on half MS agar plates. Plants were grown in a Percival growth chamber at 21°C under 16‐/8‐hour light/dark cycle with 70% humidity.

### 2.2 Phylogenetic analysis

Physcomitrella XyG‐related GTs were identified using AtMUR3 as a template for a BlastP search of the Physcomitrella phytozome database (version 10.1). Alignment of the XyG‐related GT47 proteins was achieved by MUSCLE alignment and construction of a phylogeny tree using PhyML (Dereeper, Audic, Claverie, & Blanc, 2010; Dereeper et al., 2008).

### 2.3 Gene constructs and plant transformation

Physcomitrella candidate genes were amplified either by PCR from genomic DNA or by RT‐PCR from total RNA extracted from 1‐week‐old protonemal tissue and 1‐month‐old gametophytes of Physcomitrella. Primer sequences used for cloning are listed in the additional Table S2. The amplified genes were cloned into the expression vector pORE‐E4 containing a 35S promotor for ubiquitous expression (Coutu et al., 2007), which was transformed to *Agrobacterium tumefaciens* strain GV3101, and subsequently transformed to Arabidopsis via the floral dip method (Clough & Bent, 1998). Three generations of transgenic *PpXDT* and *PpXLT2* plants were selected on half MS agar (0.8%) plates containing 60 μg/ml kanamycin. Germinated seedlings were then moved to soil for continuous growth. T3 plants were used for phenotypic analysis growth habit and root length.

### 2.4 Analysis of xyloglucan

XyG oligosaccharides were extracted from leaf tissue of Arabidopsis Col‐0, *Atmur3.1 xlt2*,* PpXDT mur3.1 xlt2, PpXLT2 mur3.1 xlt2*, and *Selaginella Kraussiana* by alcohol insoluble residue (AIR) preparation followed by xyloglucanase digestion (Pauly et al., 1999) and subsequent XyG oligosaccharide profiling by MALDI‐TOF MS and HPAEC‐PAD as described (Jensen et al., 2012; Schultink et al., 2013).

### 2.5 Purification of xyloglucan oligosaccharide XXDG

Extraction of the XyG oligosaccharide XXDG (m/z 1217) from transgenic plants was performed according to methods described in Schultink et al., 2013. However, the reduction in the oligosaccharides by sodium borohydride was performed after separation of the oligosaccharides by HPAEC‐PAD. The reduced oligosaccharides were neutralized, desalted using a ENVI‐CARB reverse phase column (Sigma Aldrich, USA) and freeze‐dried in a lyophilizer.

### 2.6 Glycosyl linkage analysis

Glycosidic linkage analysis of XyG oligosaccharides was performed as described (Schultink et al., 2013).

### 2.7 NMR analysis

Reduced oligosaccharides were dissolved in 0.3 ml of D_2_O (99.9%, company?), freeze‐dried and dissolved again in 0.3 ml of D_2_O (99.9%) containing 0.05% of 3‐(trimethylsilyl)‐propionic‐2,2,3,3‐d4 acid sodium salt. The ^1^H NMR spectra were recorded on a Bruker Avance 600 MHz NMR spectrometer equipped with an inverse gradient TXI ^1^H/^13^C/^15^N cryoprobe at 298 K. All chemical shifts were referenced relative to 3‐(trimethylsilyl) propionic‐2,2,3,3‐d4 acid (0.00 ppm for ^1^H). The NMR data processing and analysis were performed using Bruker's Topspin 3.1 software.

## 3 RESULTS

### 3.1 Identification of XyG‐related GT47 family members in the moss physcomitrella

**Figure 2:**
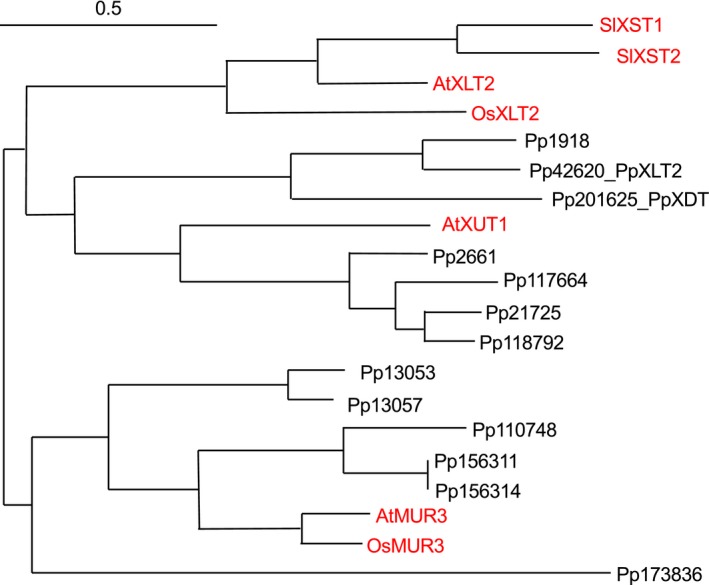
Phylogeny of XyG‐related GT47 proteins. Red font ‐ Protein sequences from known XyG:galactosyltransferases AtMUR3, OsMUR3, AtXLT2, and OsXLT2, the galacturonosyltransferase AtXUT, and the arabinofuranosyltransferases SlXST1 and SlXST2. Black font ‐ Physcomitrella proteins obtained from a BlastP search against phytozome database. PhyML tree was built by “A la Carte mode” (MUSCLE alignment and maximum likelihood computation, bootstrap = 500, scale indicated). Sl – *Solanum lycopersicum* (tomato); At – *Arabidopsis thaliana*; Os – *Oryza sativa* (rice); Pp – *Physcomitrella patens* (spreading earthmoss)

The amino acid sequence of the Arabidopsis XyG‐related GT47 family member AtMUR3 was used as a bait to identify related GT candidates of Physcomitrella present in the Joint Genome Institute database Phytozome (phytozome.jgi.doe.gov). Based on amino acid sequence homology, 13 Physcomitrella proteins were identified, which were also homologous to other, known GT47 XyG‐related proteins from various species (AtXLT2, AtXUT, OsMUR3, OsXLT2, and SlXST; Figure 2). Of the 13 Physcomitrella proteins, six members grouped closely in a MUR3 subclade. The other seven Physcomitrella proteins fell into the XLT2 subclade that also included XST and XUT. Based on the location in the protein phylogenetic tree, nine nonredundant proteins were chosen for further investigation (Pp1918, Pp42620, Pp201625, Pp2661, Pp21725, Pp173836, Pp156311, Pp110748, and Pp13057).

### 3.2 Functional complementation in arabidopsis and characterization of XDT

**Figure 3:**
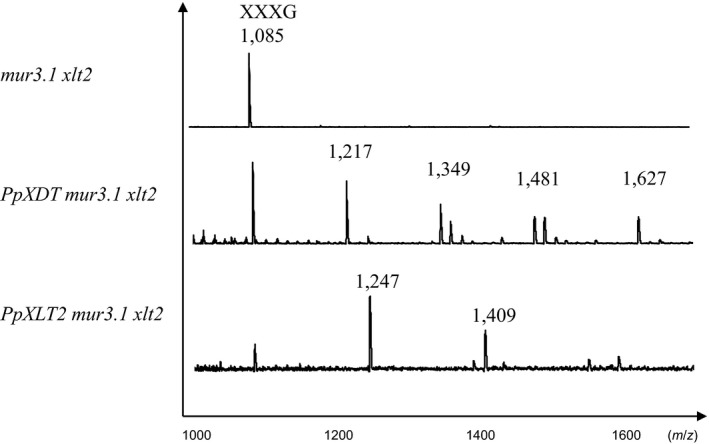
XyG oligosaccharide mass profiling by MALDI‐TOF MS. XyG oligosaccharides derived from leaf tissue of the Arabidopsis double mutant *mur3.1 xlt2*, transgenic lines expressing *PpXDT* or *PpXLT2* in *Atmur3.1 xlt2*. Numbers indicate m/z, and their potential structure is shown in Table S1. m/z 1085 represents the known XyG oligosaccharide structure XXXG

To assign GT functions to the nine selected Physcomitrella GT47 family members, heterologous expression of individual proteins in the Arabidopsis double mutant *Atmur3.1 xlt2* was pursued. XyG derived from the various complemented Arabidopsis plants was analyzed by oligosaccharide mass profiling (OLIMP) (Gunl, Kraemer, & Pauly, 2011), whereby XyG was solubilized from wall materials using a XyG‐specific endoglucanase, and the resulting oligosaccharide mixture was analyzed by MALDI‐TOF mass spectrometry (Figure 3). The OLIMP profile of untransformed *Atmur3.1 xlt2* mutant plants shows the occurrence of a single oligosaccharide motif with a m/z of 1,085 representing the XyG oligosaccharide XXXG consisting only of the glucan backbone with xylosyl moieties but no further substitutions. This OLIMP profile was retained when seven of the Physcomitrella genes were constitutively expressed in Arabidopsis *mur3.1 xlt2* suggesting that in Arabidopsis these genes may not be involved in XyG biosynthesis. However, other possibilities of nonfunctionality include the presence, instability, and mislocalization of these expressed proteins.

**Figure 4:**
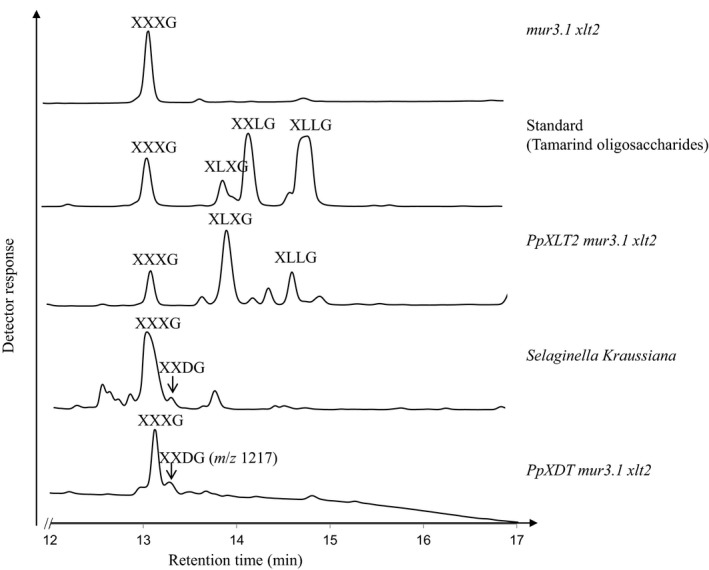
XyG oligosaccharide separation by HPAEC‐PAD. Peaks were assigned based on retention times from published work (Schultink et al., 2013; Hsieh & Harris, 2012; Megazyme Inc., Ireland) as well as assignment based on mass spectrometry. The fractions containing m/z 1217 (XXDG) were collected and further analyzed by glycosidic linkage analysis (Table 1) and NMR spectroscopy (Figure 5)

**Figure 5:**
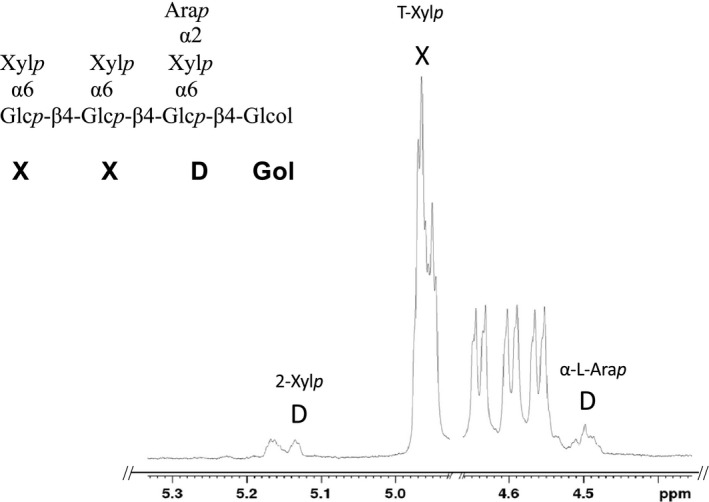
Anomeric region of the ^1^H NMR spectra of the reduced XyG oligosaccharide XXDGol contaminated with XXXGol. ^1^H NMR peaks were labeled according to CCRC (ccrc.uga.edu) database and the literature (Pena et al., 2008). The location of each of these residues in specific side chains (X or D) is indicated

Nevertheless, expression of *Pp201625 (PpXDT)* in *Atmur3.1 xlt2* resulted in a XyG that contained an oligosaccharide of m/z 1,217 representing an oligosaccharide consisting of 4 hexoses and 5 pentoses (H4P4) not found in Arabidopsis WT or *Atmur3.1 xlt2*. This result indicates that this GT affects XyG biosynthesis and is responsible for adding an additional pentosyl residue to XXXG in *Atmur3.1 xlt2* (Figure 3). Moreover, five additional XyG oligosaccharides were observed when *PpXDT* was expressed in *Atmur3.1 xlt2* (Figure S1). These ions correspond to oligosaccharide structures such as m/z 1,349 (H4P5), 1,363 (H4P4 with an additional deoxysugar, likely to be fucose), 1,481 (H4P6), 1,495 (H4P5 with an additional fucose), and 1,627 (H4P6 with a fucose). Mass spectrometry does not indicate the identity the added pentose nor where it would be attached. To determine the fine structure of the dominant novel XyG oligosaccharide (m/z 1,217), the oligosaccharide was enriched by subjecting the XyG oligosaccharide mixture obtained from wall material of *Pp201625 Atmur3.1 xlt2* to high‐performance anion‐exchange chromatography with pulsed amperometric detection (HPAEC‐PAD; Figure 4). Oligosaccharide(s) with a molecular mass of m/z 1,217 eluted at ~13.2 min and were collected for further analysis. Some impurities of the XyG oligosaccharide XXXG were present in the collected fraction due to its adjacent elution. The retention time of the novel oligosaccharide was found to be the same as a well‐characterized XyG oligosaccharide isolated from *S. kraussiana* (Hsieh & Harris, 2012), termed XXDG, a XyG oligosaccharide containing an arabinopyranosyl residue (Figure 4). The isolated/enriched oligosaccharide with a m/z of 1217 isolated from *Pp201625 Atmur3.1 xlt2* was subjected to glycosidic linkage analysis (Table 1). The presence of t‐Ara*p* and 2‐Xyl*p* in an equal ratio supports the hypothesis that Ara*p* is attached to Xyl*p* at *O*‐2, thus representing the structure of XyG D side chain. No t‐Ara*f* was detected. To gain further insights into this structure, ^1^H NMR was performed (Figure 5, Additional Figure S1). Based on previously observed chemical resonances (Pena et al., 2008), the data confirmed the presence of an anomeric signal of an α‐linked arabinopyranosyl residue (chemical shift of 4.488 ppm). In addition, the corresponding substituted 2‐α‐Xyl*p* residue was identified with a chemical shift of 5.133 ppm. The observed chemical shifts are therefore consistent with the presence of the oligosaccharide XXDG (Figure 5). As *Pp201625* plays a role in transferring arabinopyranose to XXXG in the Arabidopsis mutant *mur3.1 xlt2* to generate a XyG D side chain, it was termed XyG D side chain Transferase (XDT).

**Table 1:** Glycosidic linkage analysis of XyG oligosaccharide fraction with a m/z 1217 (with contamination of XXXG) from leaf walls of *PpXDT Atmur3.1 xlt2*

Sugar moiety	Abundance (%)
t‐Ara*p*	2.88
t‐Xyl	38.20
2‐Xyl	2.88
6‐Glc	14.57
4‐Glc	13.29
4,6‐Glc	28.18

2‐Xyl, 2‐linked xylose; 4,6‐Glc, 4,6‐linked glucose; 4‐Glc, 4‐linked glucose; 6‐Glc, 6‐linked glucose; t‐Arap, terminal arabinopyranose; t‐Xylp, terminal xylopyranose.

### 3.3 Heterologous expression of *PpXLT2*

The Physcomitrella GT47 family also contains Pp42620, a protein that phylogenetically belongs to the AtXLT2 subclade (Figure 2). Expression of *Pp42620* in *Atmur3.1 xlt2* resulted in the generation of various XyG oligosaccharides, when the transgenic plants were analyzed by oligosaccharide mass profiling. The oligosaccharides with a m/z of 1,247 represent XXXG plus an additional hexose—the minor new oligosaccharide with a m/z of 1,409 represents XXXG plus 2 hexoses (Figure 3). To determine the fine structure of the dominant XyG oligosaccharide (m/z 1,247), the XyG oligosaccharide mixture generated from wall materials of *Pp42620 Atmur3.1 xlt2* was analyzed by HPAEC‐PAD. Compared to previously published data (Kooiman, 1961) and tamarind XyG oligosaccharide standards, the novel oligosaccharide exhibited the same retention time as the tamarind XyG oligosaccharide XLXG (Figure 4). Oligosaccharides with the retention time of XXLG and the double galactosylated XLLG were also present suggesting that *Pp42620* exhibits mainly XLT2 activity in Arabidopsis, hence its name PpXLT2, and some additional MUR3 galactosyltransferase activity.

### 3.4 Growth phenotypes of *PpXDT Atmur3.1 xlt2* and *PpXLT2 Atmur3.1 xlt2*

**Figure 6:**
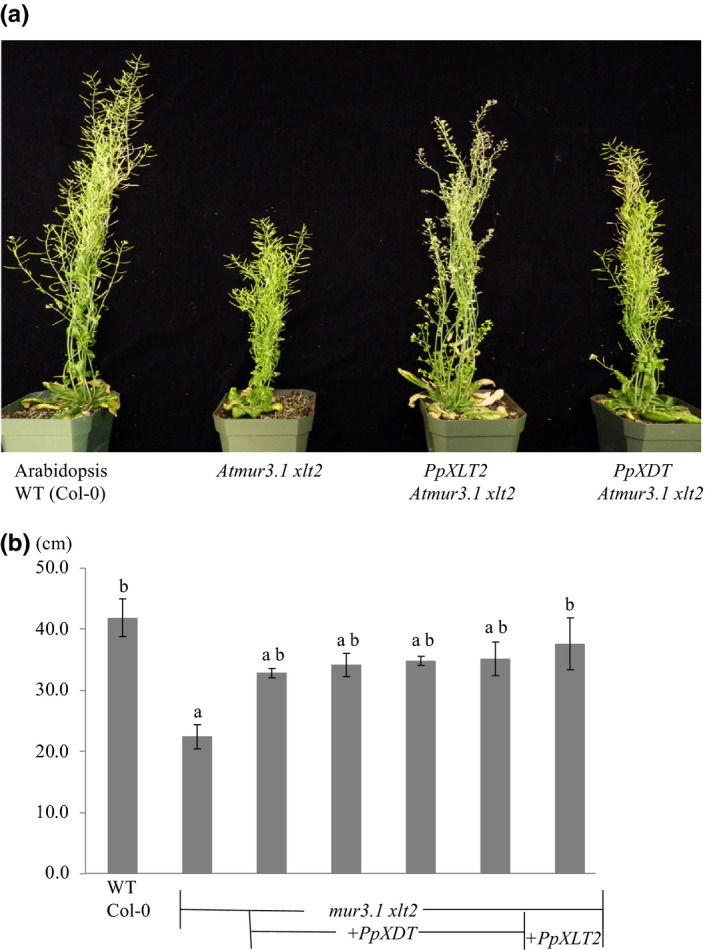
(a) Growth habit of 8‐week‐old Arabidopsis plants. (b) Height of Inflorescence stems of 8‐week‐old Arabidopsis plants. Four independent transformants of *PpXDT Atmur3.1 xlt2* (*n* = 3, each), *PpXLT2 Atmur3.1xlt2* (*n* = 4), and *Atmur3.1 xlt2* (*n* = 6) were compared to *Col‐0* (*n* = 5). ANOVA analysis was performed and subjected to a Tukey's test. Error bars indicate standard error. Small letters indicate similarity based on *p* < .05

**Figure 7:**
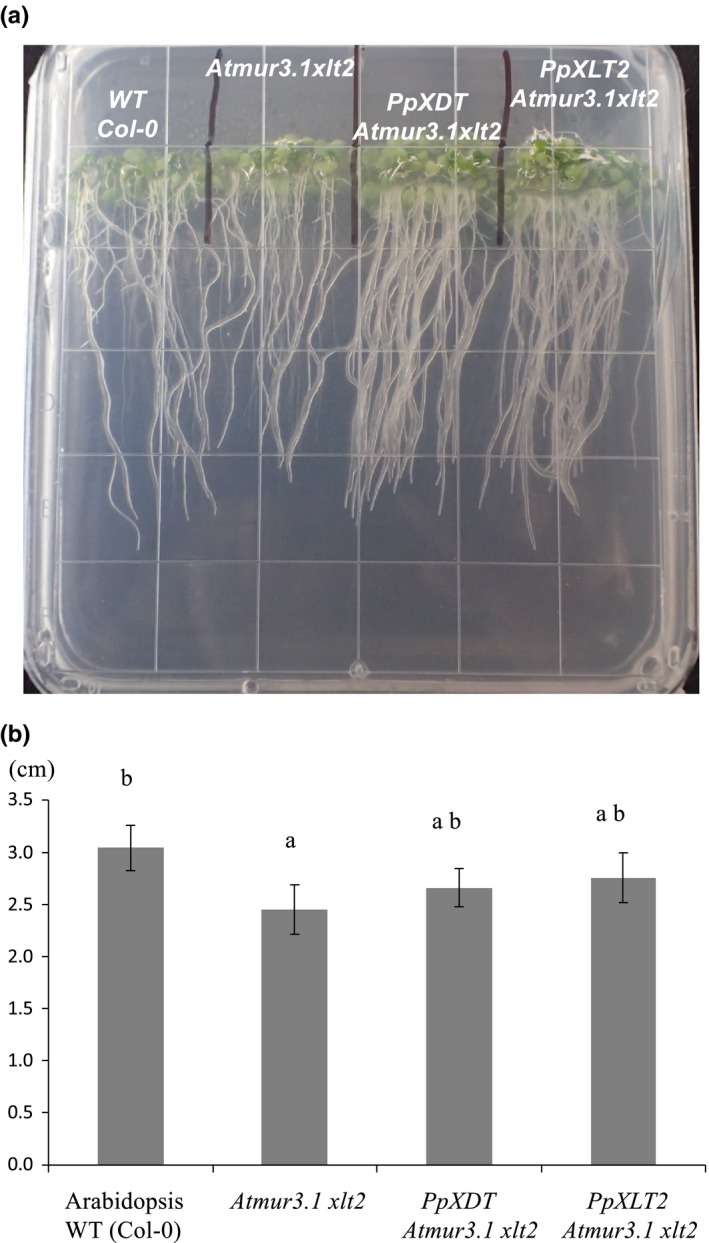
Root growth of Arabidopsis plants. (a) Root habit (b) Root length of 8‐day‐old seedlings, *n* = 10. ANOVA analysis was performed and subjected to a Tukey's test. Error bars indicate standard error. Small letters indicate similarity based on *p* < .05

The structure of XyG has been shown to affect vegetative growth. For example, the Arabidopsis double mutant *mur3.1 xlt2* containing XyG entirely composed of XXXG units exhibits dwarfism (Liu et al., 2015; Schultink et al., 2013) (Figure 6). When *PpXDT* and *PpXLT2* were expressed in *Atmur3.1 xlt2* using a constitutive promotor, vegetative (stem) growth was restored to nearly normal heights in most of the lines (Figure 6a,b). The Arabidopsis double mutant *mur3.1 xlt2* exhibits also shorter roots (Pena et al., 2012) (Figure 7a,b). Expression of Pp*XDT* and *PpXLT2* in the double mutant leads to root growth that is not significantly different than Arabidopsis WT.

## 4 DISCUSSION

### 4.1 Identification of a XyG:arabinopyranosyltransferase (XDT)

The xylosyl residue of XyG is often substituted at the *O*‐2 position with a variety of glycosyl residues including galactosyl, galacturonosyl, xylopyranosyl, arabinofuranosyl, or arabinopyranosyl moieties (Pauly & Keegstra, 2016). Using in vitro assays, loss‐of‐function mutants and functional complementation approaches in Arabidopsis have led to the successful identification of many of the responsible GTs including two galactosyltransferases, MUR3 and XLT2 (Jensen et al., 2012; Madson et al., 2003), a galacturonsyltransferase XUT1 (Pena et al., 2012), an arabinofuranosyltransferase XST (Schultink et al., 2013) and as identified and characterized in this study an arabinopyranosyltransferase XDT.

The availability of the Physcomitrella genome (Rensing et al., 2008) allowed us to identify this XyG:arabinopyranosyltransferase PpXDT. Expression of the *PpXDT* gene led to synthesis of the XyG D side chain in the Arabidopsis double mutant *mur3.1 xlt2* as evidenced by XyG analysis by MALDI‐TOF MS, HPAEC‐PAD, linkage analysis, and NMR. When a MUR3 ortholog from rice is expressed in *Atmur3.1 xlt2* XyG does not only become galactosylated, it also becomes fucosylated (Liu et al., 2015) as the galactosylated side chain L is the required acceptor substrate for the XyG:fucosyltransferase (Vanzin et al., 2002) resulting in the F side chain. Here, expression of *PpXDT* resulted not only in arabinosylated side chains, but also to a much lesser extent in side chains containing an additional deoxyhexose. These data are consistent with the occurrence of a fucosylated D side chain termed E, which has been observed in XyG derived from *Equisetum hyemale* and *S. kraussiana* (Hsieh & Harris, 2012; Pena et al., 2008). The Arabidopsis XyG:fucosyltransferase AtFUT1/AtMUR2 is apparently not only able to transfer fucosyl residues to galactosyl but also arabinopyranosyl residues. Similar to previous reports (Hsieh & Harris, 2012; Pena et al., 2008), acetylated versions of the D side chain were not observed, indicating that the Arabidopsis XyG:*O*‐acetyltransferase AtAXY4/AtAXY4L (Gille & Pauly, 2012) specifically adds acetyl substituents to galactosyl residues.

The galactosyltransferases AtMUR3 and AtXLT2 act regiospecifically on a particular xylosyl residue to generate XXLG or XLXG, respectively. The expression of *PpXDT* in Arabidopsis also led to XyG oligosaccharides contain additional pentoses, besides arabinopyranose. Although the nature and position of these additional pentosyl residues remain to be determined, it seems clear that XDT is more promiscuous in nature than MUR3/XLT2. The enzyme could potentially transfer other pentoses, such as arabinofuranoses. A more likely scenario is that XDT might be able to add arabinopyranoses to different positions on XyG resulting not only in the XXDG oligosaccharide (m/z 1,217, Table S1) but potentially also XDXG (m/z 1,217), XDDG (m/z 1,349), DDDG (m/z 1,481), and their fucosylated versions XXEG (m/z 1,363), XDEG (m/z 1,495), and DDEG (m/z 1,627). As ions of all these oligosaccharides were present when *PpXDT* was expressed in the *Atmur3.1 xlt2* mutant (Table S1), PpXDT does not seem to act regiospecifically.

### 4.2 Functional conservation of XyG‐related genes in the GT47 Family

The GT47 family is a large carbohydrate‐active enzyme (CaZy) family involved in cell wall biogenesis containing a subclade represented by the XyG:galactosyltransferase AtMUR3 (Madson et al., 2003) and includes AtXLT2, another XyG:galactosyltransferase (Jensen et al., 2012). The identified Physcomitrella XyG:arabinopyranosyltransferase PpXDT also belongs to this subclade, as it forms the same glycosidic linkage on the same acceptor substrate albeit utilizing a different donor substrate (UDP‐L‐arabinopyranose). Taken together with the characterized functions of other GT47 members such as AtXUT1 from *Arabidopsis* and SlXST from tomato, members of the GT47 MUR3 subclade in land plants have evolved in transferring a glycosyl moiety to the xylosyl residue of XyG at *O*‐2.

Within this clade, another functional XyG GT was identified in Physcomitrella, PpXLT2. Analysis of the XyG present *Atmur3.1 xlt2* expressing *PpXLT2* resulted in the occurrence of XLXG indicating that PpXLT2 in *Arabidopsis* can carry out the same function as AtXLT2, in addition to also forming XXLG and its fucosylated version XXFG. Thus, unlike AtXLT2 from Arabidopsis (Jensen et al., 2012), SlXLT2 from tomato (Schultink et al., 2013), and OsXLT2 from rice (Liu et al., 2015), the Physcomitrella ortholog PpXLT2 also exhibits MUR3 activity, albeit with a regiospecific preference of an XLT2. While the function of XLT2 is functionally conserved across land plants including bryophytes, the strict regioselectivity of this GT has apparently evolved later, as it has only been observed in angiosperm species investigated to date.

### 4.3 XyG side chains impact aerial and root growth

The Arabidopsis mutant *mur3.1* contains a point mutation in the *AtMUR3* gene that renders it inactive (Madson et al., 2003). As a result, XyG in this mutant does not contain the XXLG oligosaccharide, but retains the galactosylated XLXG motive. Mutant plants show normal plant growth except for minor effects in trichome morphology (Madson et al., 2003). However, the double mutant *Atmur3.1 xlt2*, whose XyG does neither contain XXLG nor XLXG oligosaccharides, but consists entirely of XXXG oligosaccharides displays a dwarfed phenotype (Kong et al., 2015). Complementing this mutant with various XyG GT47 genes can rescue the growth defect. This rescue has been observed with the expression of *MUR3* and *XLT2* from a variety of species such as from rice *OsXLT2* (Liu et al., 2015), tomato *SlXLT2* (Schultink et al., 2013), or as shown here from Physcomitrella *PpXLT2*. Moreover, arabinofuranosylation by expressing SlXST (Schultink et al., 2013) and as shown here arabinopyranosylation through PpXDT also restores the phenotype of the double mutant, not only the growth of vegetative tissue, but also root growth. This indicates that galactosylation or the occurrence of the L side chain is not required for normal growth, but that alternative substitutions such as arabinofuranosylation and arabinopyranosylation resulting in the S and D side chains, respectively, suffice for normal plant growth. It is known that XyG that consists only of XXXG self‐aggregates and precipitates in vitro (Buckeridge, 2010; Gibeaut, Pauly, Bacic, & Fincher, 2005). Such precipitation of nongalactosylated XyG in the *Atmur3.1 xlt2* mutant might occur already during its biosynthesis in the Golgi apparatus impacting the function of the endomembrane system. Indeed, the Arabidopsis *mur3.3* mutant, an insertional knockout mutant, exhibits severe dwarfism with a concomitant aggregation of endomembranes and intracellular accumulation of polymers (Kong et al., 2015). However, when the *Atmur3.3* mutant is crossed with the XyG‐lacking *Atxxt1 xxt2* mutant the resulting mutant plant exhibits not only again a normal growth phenotype but also a normal endomembrane morphology. XyG is still lacking in these plants. Hence, a structurally aberrant XyG with low or lacking galactosylation is detrimental to plant development, whereas a lack of XyG is not.

## CONFLICT OF INTEREST

The authors declare no conflict of interest.

## AUTHORS CONTRIBUTIONS

LZ performed the experiments, analyzed the results, and wrote the manuscript. MD performed the NMR experiments and wrote the manuscript. MP conceived the work, analyzed the data, and wrote the manuscript.

## ETHICS APPROVAL AND CONSENT

The leaf samples used in this study were collected from the growth chamber in our laboratory and from the botanical garden at the University of California, Berkeley with permission of the curator. The experimental research was undertaken in accordance with local guidelines. For access to the plants, please contact the corresponding author.
