# Supplementary material for: Identification of an arabinopyranosyltransferase from Physcomitrella patens involved in the synthesis of the hemicellulose xyloglucan
Source: Plant Direct. 2018 Mar 13;2(3):e00046. doi: 10.1002/pld3.46 (PMC6508525; doi:10.1002/pld3.46)
Supplement: Supplementary file 1 [file PLD3-2-e00046-s001.pdf]

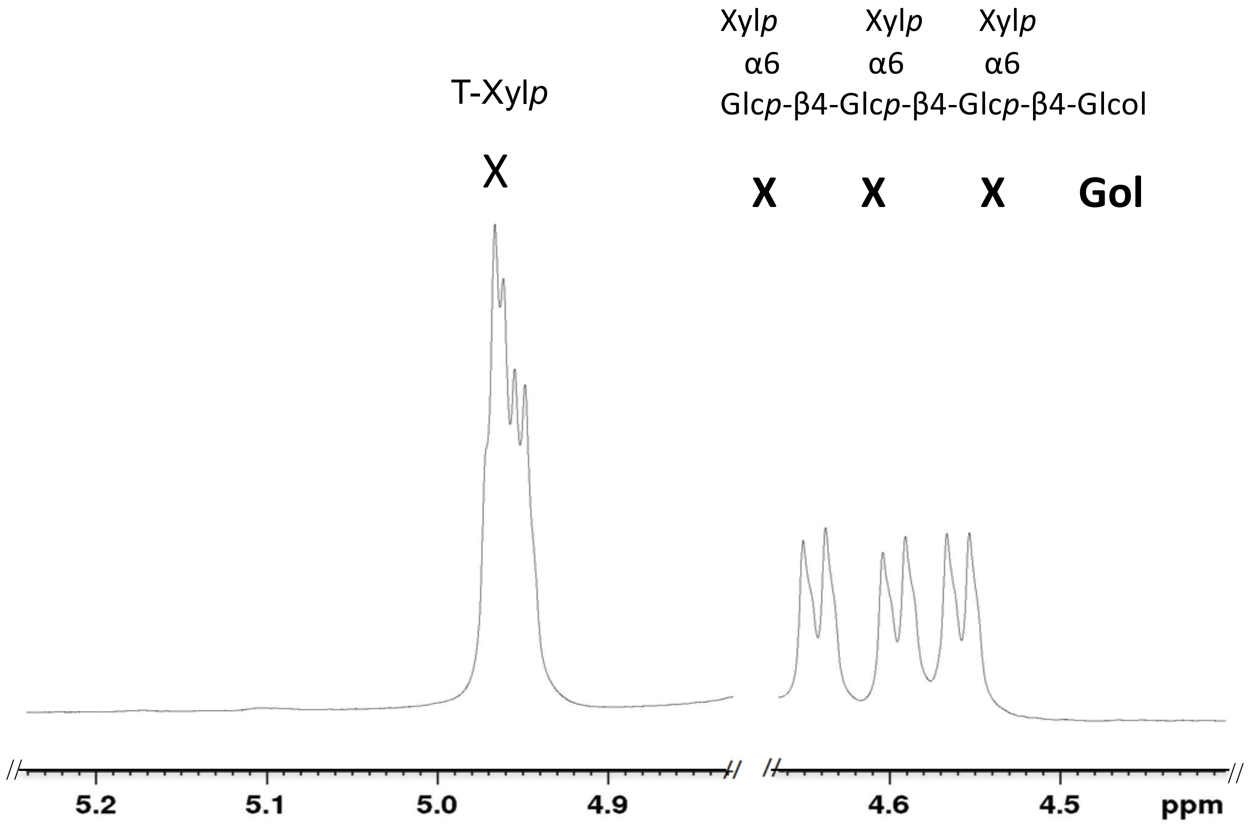

Figure S1. Anomeric region of the ^{1}H NMR spectra of the oligosaccharide XXXG obtained from *Atmur3.1 xlt2*. ^{1}H NMR peaks were labelled according to CCRC (ccrc.uga.edu) database and the literature (Pena et al., 2008). The location of each of these residues in the specific sidechain (X) is indicated.

Table S1. The XyG oligosaccharide composition based on ions (m/z) seen in Fig. 3.

	Ions (m/z)	Oligosaccharide composition	Predicted Oligosaccharide Structure [a]
PpXDT mur3.1 xlt2	1085	H4P3	XXXG
	1217	H4P4	XXDG/XDXG
	1349	H4P5	XDDG
	1363	$H4P4H_{de}1$	XXEG/XEXG
	1481	H4P6	DDDG
	1495	$H4P5H_{de}1$	XDEG/XEDG
	1627	$H4P6H_{de}1$	DDEG/DEDG
PpXLT2 mur3.1 xlt2	1085	H4P3	XXXG
	1247	H5P3	XXLG/XLXG
	1409	H6P3	XLLG
	1555	$H6P3H_{de}1$	XLFG
	1597	$H6P3H_{de}1Ac1$	XLFG-Ac

P - pentose; H - hexose; H_{de}, - deoxy hexose; Ac - acetate.
[a] Assuming that PpXDT only transfers arabinopyranosyl-residues

Table S2. Primers for PCR amplification of genes

Primers	Sequences
Pp_42620_exon-F-Gibson	5'- AGA ATT CGT CGA CTT TGC ATG
Pp_42620_exon-R-Gibson	5'- AGT AAA AGG TAC CGA GCT TCA
Pp_201625_CDS_Gibson-F	5'- AGG GAT ATC ACT AGT CAA CAA
Pp_201625_CDS_Gibson-R	5'- CAA AAC CCA CCG GAT ATG GGG
Pp201625_CDS-R	5'- TCA ACA ACC ATC CGT GAC CTT
Pp201625_CDS-F	5'- ATG GGG TAT GCA CCG CAA T
Pp11_copy# FWD Set 1	5'- TTC GTC CGC TCG GTG GGT AAT TT
Pp11_copy# REV Set 1	5'- GTT CTC AAC GGC CGA GAT CCA TT
Pp12_copy# FWD Set 5	5'- TGC ATC CGA CCG AAG TCG AAA C
Pp12_copy# REV Set 5	5'- ACA GAG CTC CGT GGA GCA AAT G
