# Supplementary material for: Identification of an arabinopyranosyltransferase from Physcomitrella patens involved in the synthesis of the hemicellulose xyloglucan
Source: Plant Direct. 2018 Mar 13;2(3):e00046. doi: 10.1002/pld3.46 (PMC6508525; doi:10.1002/pld3.46)
Supplement: Supplementary file 2 [file PLD3-2-e00046-s002.pdf]

Dear editor,
We are delighted that the reviewers appreciated the discoveries made in the manuscript and hereby resubmit an improved version that addresses their concerns.

Reviewer 1:
Fig2 - please write out in full which species are represented by the abbreviations - two letters really is not enough. It took this reviewer a while to work out that your Sl was tomato.

Implementation according to the reviewer's suggestions.

Since multiple species are referred to in this article, I think it would be clearer if all genes were preceded by their species two letter code e.g. Atmur3 Atxlt2. However, that may be an editorial/journal style decision.

Implementation according to the reviewer's suggestions.

There is a mixture of nomenclature in the text: Physcomitrella, Physcomitrella and Physcomitrella patens. In my opinion it should be 1st use Physcomitrella patens and then either P. patens (better) or Physcomitrella.

Based on the comments made by the reviewer, In the first instance *Physcomitrella patens* was used, in the following instances Pp for the genes and Physcomitrella only was used.

Figure 5/S1 - could you find a better way to present your putative structure? Perhaps some color for either the one letter code or the a,b,c? Since the alditol nomenclature isn't so common, I would prefer to see this explained in the legend or methods.

The representation was changed based on Pena et al., 2008 in both Figs.

Some minor grammatical errors need to be addressed throughout prior to publication, noticeably in the discussion. e.g. Line 185 and Line 194 "act regiospecific" should be "act regiospecifically", Line 210 "its regioselectivity of this" should be "the regioselectivity of this", Line 214 "Arabidopsis...gene and renders it inactive" should be "The Arabidopsis....gene that renders it inactive", Line 215 "but still the" should be "retains the" OR "still contains the" etc. This is not an exhaustive list.

The semantics pointed out here were changed according to the reviewer's suggestions. In addition, the manuscript was read by a native English speaker.

The plant phenotyping data is not sufficiently described. How many plants were measured? What generation? How many biological replicates of independently grown plants? What do the error bars represent? Very minor - why in Figure 7 is the root growth data not under the representative image ie WT below WT (and the nomenclature not the same ie italicized mur3 etc). This all makes it simpler for the reader to interpret.

The data description was added in the legend and material and methods. The figure was modified to align the pictures with the graph bars as closely as possible.

Reviewer 2:
1. Ln. 110 states that seven of the Pp genes 'are not involved in XyG biosynthesis in Arabidopsis'. However, without data showing that the transgenes are in fact expressed in

Arabidopsis such a conclusion cannot be made. Even if the transcripts are present, one can still question if the proteins are present.

We agree with the reviewer and have modified the section.

2. In ln. 116 or 122, it should be explicitly stated that the m/z 1217 oligosaccharide is the one called 'H4P4'.

An amendment has been made according to the reviewer's suggestion.

3. A major issue is the claim in the discussion (ln 194 and 210) and that the Pp enzymes are not regiospecific. This does not quite match the data. In Fig. 3 it is clearly seen that the XLXG fragment is much more abundant than XXLG. I am not sure about XXDG in Fig. 3, since there is no standard for XDXG and the peaks are small anyway. But it seems to me that a more correct statement is to say that the Pp enzymes have regioselective preference but that regioselectivity is not 100% strict as observed in the angiosperm enzymes that have been studied so far. One should probably also consider that not very many angiosperm enzymes have been studied, so who knows if the strict regioselectivity is seen in all the angiosperm enzymes.

We agree with the reviewer that the phrase "regioselective preference" would be a more correct term. We have modified the discussion accordingly

4. Related to this comment, there is no quantification of the different oligosaccharides. According to the heading, Table S1 would show relative abundance of the ions, but that information is actually not in the table.

Initially the ion abundance was present in the table, but was deleted as further analyses would be required to ascertain these quantities. The text of table S1 has been changed accordingly.

5. In ln. 250 there should be a reference for the pORE-E4 vector. It might also be useful to mention which promoter that is present in this vector.

The vector includes the 35S promotor, which we included in the material and methods.

6. Table S2 has primers for GAPDH. There is no indication what they were used for. Perhaps the expression analysis that I am mentioning in comment 1?

The primers were deleted.

7. Fig. 6 and 7. What are the error bars?

Error bars represent standard errors, which has now been incorporated into the legend

8. Fig. 6 and 7. ANOVA is not adequately described. The small letters over the bars presumably indicate similarity based on some threshold p level (p{greater than or equal to} 0.05 for same letter?), but it is not stated. Also, how were multiple comparisons made? ANOVA does not automatically give the information required to put the small letters over the bars. Multiple comparisons can be done in different ways (e.g. Tukey's test), so it is important to specify how.

We thank the reviewer for this comment and included more information in the legend of Fig.s 6 and 7

9. Figure 2. Bootstrap values should be calculated and shown.

Bootstrap values have been calculated and a scale has been added to the figure.

10. There are many commas missing, which makes it difficult to read in some places (e.g. In 98, 103, 208)

The manuscript has been read by a native English speaker and hopefully the commas are now all placed.
